# Near-Infrared Spectroscopy for the Single-Kernel Analysis of Sorghum Protein Content

**DOI:** 10.3390/s26102936

**Published:** 2026-05-07

**Authors:** Princess Tiffany D. Mendoza, Paul R. Armstrong, Erin D. Scully, Xiaorong Wu, Kamaranga H. S. Peiris, Scott R. Bean, Kaliramesh Siliveru

**Affiliations:** 1 Department of Grain Science and Industry, Kansas State University, Manhattan, KS 66506, USA; kaliramesh@ksu.edu; 2 Agricultural Research Service, Center for Grain and Animal Health Research, USDA, Manhattan, KS 66502, USAerin.scully@usda.gov (E.D.S.); shawn.wu@usda.gov (X.W.);

**Keywords:** near-infrared spectroscopy, sorghum, protein content, chemometrics

## Abstract

Protein content is an important quality trait in sorghum that influences breeding approaches, end-use applications, and market value. Influenced by genetic, agronomic, and environmental variability, sorghum is characterized by its wide variation in composition, which may also be evident in kernels from the same sample. This study developed and evaluated a method for a non-destructive and rapid prediction of protein content in individual sorghum kernels using single-kernel near-infrared spectroscopy (SKNIR). Applying different pre-processing techniques to the spectra collected from intact kernels, the calibration models were developed using partial least squares regression and the reference protein content values obtained from the LECO combustion method. The best model was obtained using multiplicative scatter correction as pre-processing, resulting in a standard error of prediction of 0.83% and a relative predictive determinant of 3.40. These were indicative of the good predictive ability of the model and the instrument to be applied in quality control and sorting applications. These results highlight the potential of SKNIR to capture the inter-kernel variability in sorghum protein content and enhance screening for grain quality in breeding and grain processing.

## 1. Introduction

Sorghum (*Sorghum bicolor* L.) is a global cereal valued for its drought tolerance and agronomic adaptability [1]. It has been increasingly recognized for its versatility and rich nutritional profile [2]. In the United States, the top sorghum producer with 8.73 million metric tons produced in 2024/2025, sorghum has historically been predominantly used for animal feed, but its use in human food is growing [3,4]. Along with its diverse range of uses, sorghum has a broad genetic variability, as reflected in its substantial heterogeneity in grain composition, including its protein content that ranges from 6 to 20% [5]. Sorghum typically contains protein content that is comparable to wheat and higher than rice and corn [6]. Protein content is one of the major quality parameters that influence the end-use quality of sorghum. Obtaining an accurate measurement of sorghum protein content for breeders and grain processors is vital for optimizing the nutritional quality and end-use value of sorghum.

Traditional laboratory methods for protein content determination in grains are slow, labor-intensive, and costly. Alternative rapid and non-destructive methods have been utilized and continuously developed for efficient quality analysis, particularly for large sample sizes. One of these methods is near-infrared (NIR) spectroscopy, an analytical technique that has been extensively applied in grains and cereals. NIR spectroscopy works by measuring the light absorption of the sample, dominated by overtones and combination frequencies of O-H, C-H, and N-H bonds that can be related to the sample composition [7,8]. In grains, NIR spectroscopy has been useful in predicting moisture, protein, oil, fiber, starch, and ash contents. It is a standard method for the determination of protein in wheat, wheat flour, and soybeans [9,10,11,12,13].

With its high dimensionality, NIR spectroscopy is typically combined with chemometric techniques to extract relevant sample information; transform, pre-process, and analyze spectral data; and develop models for supervised or unsupervised learning. Pre-processing in chemometrics plays an important role in minimizing or removing undesirable variability, which can be caused by the instrument, experiment, and other factors [14]. Most of these pre-processing methods are for different individual functions, such as smoothing, baseline correction, normalization, scatter correction, and detrending, and can also be used in combination with two or more techniques [15,16]. Common pre-processing methods for grains and oilseeds are the standard normal variate (SNV), multiplicative scatter correction (MSC), and Savitzky–Golay first or second derivatives. These techniques may improve model predictive performance but may also cause the model performance to degrade if relevant information has been inadvertently removed [17]. The effect of the pre-processing method is evaluated during the model development by partial least squares regression (PLSR), multiple linear regression (MLR), principal component regression (PCR), support vector machine (SVM), artificial neural network (ANN), or other multivariate regression algorithms. PLSR is the most widely used among these algorithms, owing to its robustness to address spectral data collinearity and reduce data dimensionality [18]. PLSR works by relating X and Y data matrices, modeling the structure of these matrices, and typically improving the precision of the model parameters as the relevant variables (or factors) and samples are increasing [19].

Available commercial NIR spectrometers predict the composition of grain samples in bulk and obtain the average spectrum over a sample with multiple kernels. This method may mask the detection of kernels with extreme composition. Hence, it can be challenging to identify individual kernels with significant variations from the average composition within a population [20]. Such variations may be related to genetics, production environments, and grain processing conditions relevant to breeding programs and end-use applications.

Over the years, single-seed or single-kernel instruments have been developed for NIR spectroscopy. By capturing the spectrum of a kernel at a time, a single-kernel NIR (SKNIR) spectrometer can offer more in-depth analysis of the attributes and variability within grain samples. This kernel-level resolution will allow plant breeders to examine variability across different factors, cultivars, or growing conditions, and then select specific kernels with the desired traits while preserving the kernels for future use [21]. Compared to the bulk NIR, the SKNIR has a higher spectral noise but a higher sensitivity and resolution that can cover the inter-kernel differences, such as orientation, size, surface texture, and thickness. In terms of protein content, SKNIR enables the analysis of the protein content distribution of kernels within a sample and reveals compositional heterogeneity, both of which are not available in bulk NIR. These advantages are also relevant from a grain processing standpoint, where blending and commingling may occur in the grain supply chain, and different end-use applications may have specific protein content requirements. However, in commercial trading and grain elevator operations, bulk NIR is more practically efficient to use than SKNIR.

Studies investigating the use of SKNIR have presented applications to analyze the composition of major cereals and oilseeds such as corn, soybean, and wheat [21,22,23]. Major findings were related to the higher prediction performance of the model when based on the reference composition of each kernel and the applicability of the reflectance mode in heterogeneous seeds. It was also found that pre-processing methods, specifically SNV, MSC, and derivatives, were effective in model calibrations for percent composition or relative units. These studies have shown how SKNIR can be utilized for specific end-users for large-scale and efficient phenotyping and classification functions. With real-time prediction and analysis combined with a simultaneous sorting mechanism embedded into the system, an SKNIR instrument can provide high-throughput classification according to certain protein levels or reject defects and out-of-range kernels.

Despite sorghum being one of the five major cereals in the world, its analysis using SKNIR is emerging but still limited. This might be due to its relatively smaller kernel size, which could pose issues in obtaining reference protein content and other wet chemistry analyses. The main objective of this study was to develop and evaluate a method for the rapid and non-destructive protein content prediction of individual sorghum kernels using SKNIR spectroscopy. As a grain with wide diversity in genetics, morphology, and composition, even in samples from the same population and environmental conditions, sorghum can potentially benefit from the ability of SKNIR to capture trait variability and detect non-uniformity within grain samples.

## 2. Materials and Methods

### 2.1. Sample Preparation

Sorghum samples utilized in this study were composed of hybrids and inbred lines from private seed companies and public breeding programs, harvested in 2021 and 2022, and grown at irrigated and dryland sites in the US, including Kansas and Texas, similar to the samples in the study by Peiris et al. [24]. The scope of the samples in this study was limited to US production, and no foreign sources were included. This sampling approach was intended to cover the variability across US production environments and to provide the proposed tool to the stakeholders in the US. Additional samples that will be added in the future will also be confined within this scope.

The samples were pre-selected in a previous study based on their NIR predicted bulk protein content [24] to include a diverse set of samples covering a broad range of proteins typical for sorghum. Eighty-nine samples were obtained with 5 or 10 kernels per sample and were randomly selected for model development. From the original five kernels per sample, an additional five kernels per sample were randomly selected from seven samples to allow for a better distribution of protein content among the samples. Hence, 82 and 7 samples contained 5 and 10 kernels, respectively.

### 2.2. Data Acquisition

A single-kernel NIR reflectance instrument that was developed for small kernel size at the USDA-ARS Center for Grain and Animal Health Research (Manhattan, KS, USA) was used to collect the spectra of sorghum samples. The instrument mainly consists of a vacuum pick-up wheel, a light tube with tungsten lights, a bifurcated fiber optic bundle, and a CDI 256L-1.7T1 spectrometer (Control Development, South Bend, IN, USA) equipped with a 256-element InGaAs photodiode array and 16-bit A/D converter. The instrument captures the spectrum of each kernel from 906 to 1683 nm at 1 nm resolution. An in-house application, which was developed using Visual Basic on the .NET Framework within the Visual Studio 2017 (Microsoft Corporation, Redmond, WA, USA) integrated development environment (IDE), was used to control the instrument and facilitate spectral collection.

The scanning of samples was initiated by turning on the instrument with the control switch and waiting for it to stabilize for a minimum of 30 min. Next, an instrument calibration was performed by acquiring the dark background reference (spectrum with lights turned off) and a white reference (spectrum of the illuminated tube). Each kernel was scanned three times by feeding it into the instrument while preserving its index and identity. As each kernel slid down the light tube at a speed of 4 kernel/s, the spectrum was collected. The resulting spectra for each kernel were averaged and then converted into apparent absorbance before model development. Due to instrument noise at the left and right sides of the spectra, the wavelength was reduced to 940–1640 nm before further analysis.

After spectral collection, the protein content of individual kernels was obtained by measuring nitrogen (with a 5.7 conversion factor) using a LECO FP-828p combustion analyzer (LECO Corporation, St. Joseph, MI, USA) based on AACC Approved Method 46-30.01 [25]. Seven kernels that were suspected of being outliers during reference data acquisition, due to an equipment issue, were removed from the dataset after verifying their values.

### 2.3. Data Analysis

Spectra and reference data were analyzed using Unscrambler X 10.2 software (Aspen Technology Inc., Bedford, MA, USA) and MATLAB R2024a (MathWorks, Inc., Natick, MA, USA). Pre-processing techniques (Savitzky–Golay first derivative [polynomial order: 2, smoothing points: 11], standard normal variate (SNV), and multiplicative scatter correction (MSC)) were applied to the mean raw absorbance spectra.

For sample partitioning, samples were first sorted by increasing protein content and divided into a 2/3 training set (group A) and a 1/3 test set (group B), wherein every third sample was assigned to the test set. This ensures that the training and test sets have comparable range and distribution of protein content. Then, within each sample in the training set (group A), 60% of the kernels were randomly assigned to the calibration set (group A1) and 40% to the validation set (group A2). In this kind of partitioning, the aim was to assess how the model predicts the protein content of the kernels in the validation set, which were from the same samples of the single kernels in the calibration set and shared similar inherent characteristics [22]. While the internal validation would measure the model performance within different kernels of the same sample, the held-out test set would provide an independent validation of predictive performance on samples not in the training set.

Model development was performed using PLSR with a non-linear iterative partial least squares (NIPALS) algorithm, an internal validation set, and a fixed number of factors at 15 for all of the different pre-processing techniques. The developed models were then used to predict the protein content on an independent test set. The performance of the model was evaluated using the coefficient of determination (R^2^), standard error of calibration (SEC), standard error of validation (SEV), standard error of prediction (SEP), and the ratio of performance to deviation (RPD), which is the ratio of reference standard deviation (SD) to standard error of validation or prediction. The RPD of test set prediction is the more important index, as it is from the prediction of an independent set of samples not used to develop calibration.

## 3. Results and Discussion

### 3.1. Sample Description and Spectra

**Figure 1:**
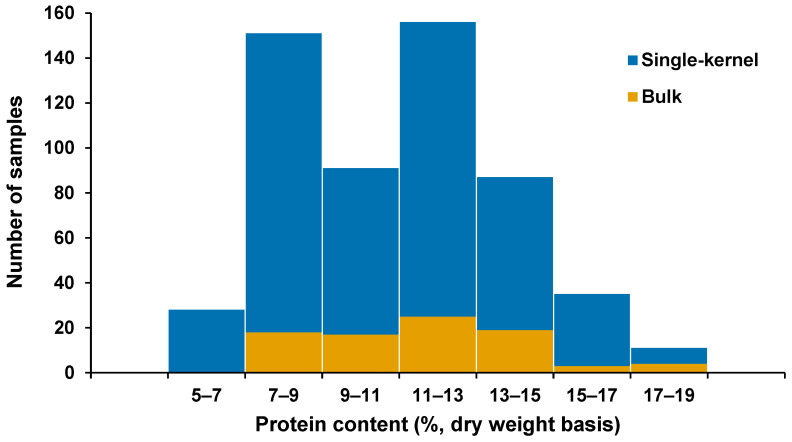
Histogram of protein content distribution in single kernel and bulk sorghum samples used in this study.

**Figure 2:**
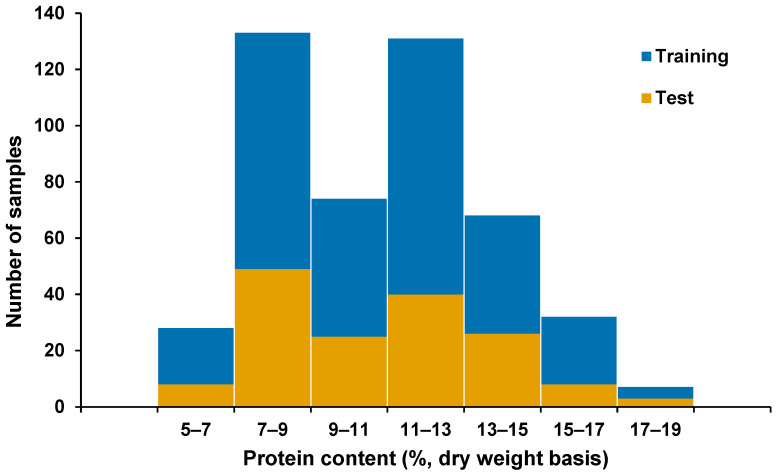
Histogram of reference protein content of sorghum single kernels used in this study, grouped according to training set (calibration, ncal = 188; validation, nval = 126) and test set (n_test_ = 159).

In this study, the reference protein content of sorghum single kernels selected for model development was from 5.35 to 18.5% (dry weight basis), with a mean of 10.84% and SD of 2.82%. Repeatability of the reference method was 0.86%, which was computed from the seven pools of 10 kernels per pool. Meanwhile, the reference protein content of the bulk sorghum samples was in the 7.18–18.81% range, with a higher mean at 11.57% and a similar SD of 2.82% compared to the single kernels. In Figure 1, which shows the reference protein content distribution of single kernels alongside the bulk samples, the variability in protein composition is evident with the wider range in single kernels than in the bulk grain samples. On the other hand, the histogram in Figure 2 illustrates the comparable distribution of the test set (n_test_ = 159) with the training set (calibration, n_cal_ = 188; validation set, n_val_ = 126).

**Figure 3:**
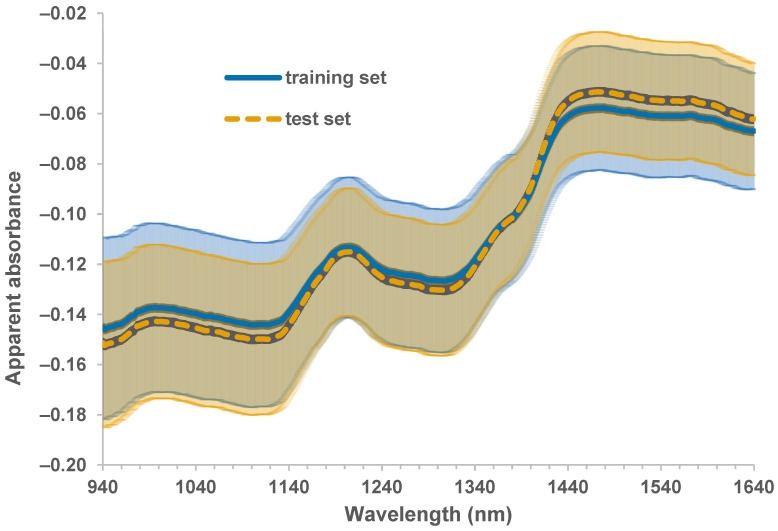
Mean raw apparent absorbance spectra (940–1640 nm) of sorghum kernels obtained from the training set (solid line) and test set (dashed line) samples using the single-kernel NIR instrument; shared area showing the standard deviation. The shaded area shows the standard deviation for each color (blue for training set and yellow for test set).

The mean apparent absorbance spectra of the individual kernels obtained using SKNIR without pre-processing are presented in Figure 3, which also shows the standard deviations for the training set and test sets in the shaded regions. It is evident from the figure that the widest spectral variability in both the training and test sets occurred at 940 nm, and it decreased until around 1410 nm.

The major peaks around 1200 nm and 1450 nm were also evident in the spectral plot presented using similar sorghum samples in bulk [24] and using different sorghum samples in another study [26].

### 3.2. Prediction of Protein Content

Table 1 presents the results from the first approach of splitting the samples into calibration and validation sets. For calibration, R^2^ and SEC were 0.94–0.97 and 0.51–0.72%, respectively. For validation, the R^2^s were 0.84 to 0.92, SEVs were 0.83 to 1.13%, and RPDs were 2.69–3.40. The values were all obtained using different pre-processing techniques at 15 factors to have a common point of reference for comparison. The model performance metrics (explained variance and standard error) were also analyzed at different numbers of factors, along with the number of factors as suggested by the Unscrambler. It was found that the standard error of validation (or prediction) could increase with a higher explained variance. Compared to raw data (0.72% SEC, 1.05% SEV, and 2.69 RPD), applying the Savitzky–Golay first derivative on the spectra improved the model in terms of calibration (SEC of 0.59%) but not validation (SEV of 1.13% and RPD of 2.50) despite increasing the number of factors to 15. SNV enhanced the model in both calibration and validation, with 0.53% SEC, 0.86% SEV, and 3.28 RPD. Our results are comparable to another study [9], which reported that SNV improved the model in predicting the protein content of single soybean seeds using a USDA light tube instrument, obtaining an RPD of 3.23 with SNV alone and 3.28 with a combination of SNV and other pre-processing techniques. In our study, the lowest SEV (0.83%) was from using MSC, which resulted in the highest RPD of 3.40. This finding is similar to other studies on protein content prediction in grains, which reported that MSC enhanced the model performance [22] and could be better than SNV [24]. Still, the performances of models with SNV and MSC were comparable, the standard errors were less than 1%, and the RPD values were within the range useful for screening, such as in breeding programs. Using either SNV or MSC could reduce the scattering effects and variance caused by light or kernel size.

**Figure 4:**
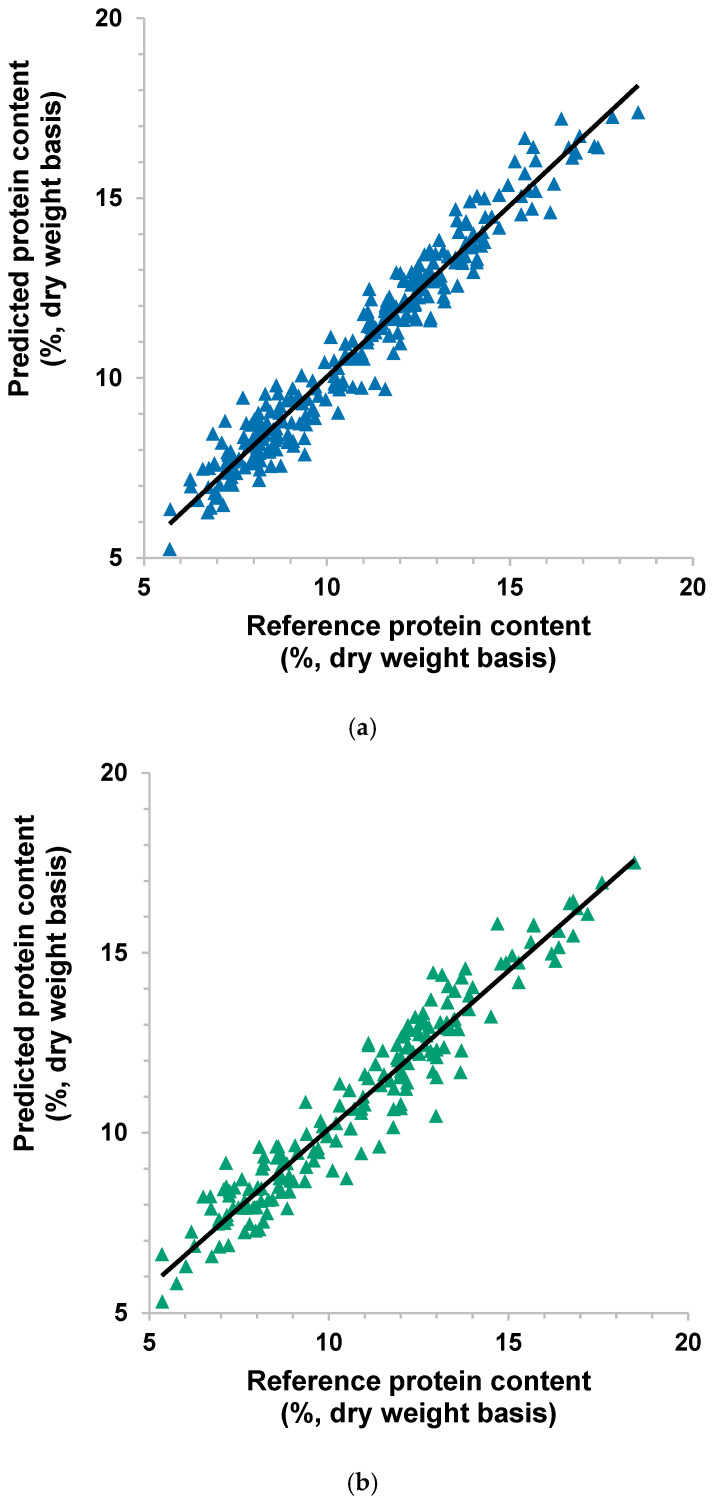
Scatterplots of reference and predicted % protein content in single sorghum kernels with multiplicative scatter correction as pre-processing: (**a**) calibration; (**b**) validation (the line is the regression line).

Using the model with MSC as pre-processing, the scatterplots in Figure 4 depict the relationship between the reference and predicted protein content of sorghum single kernels in the calibration and validation sets. More data points were slightly scattered in Figure 4b (validation) than in Figure 4a (calibration), which was also evident in the higher standard error in validation than in calibration.

**Figure 5:**
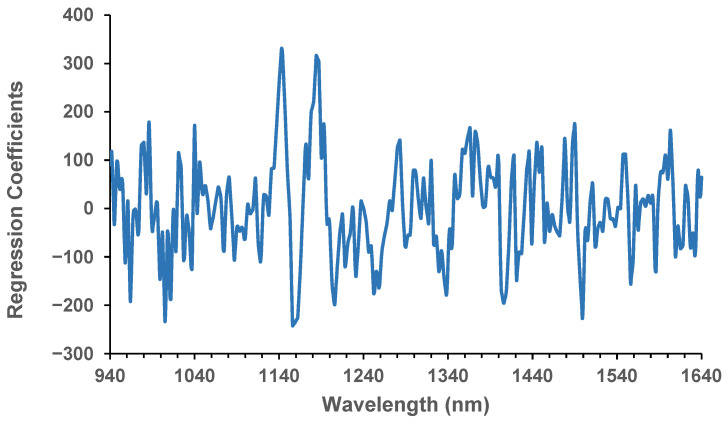
PLS regression coefficients for single-kernel sorghum with pre-processing using multiplicative scatter correction.

Since the spectra in the validation set were from different kernels but the same samples in the calibration set, this might indicate that other factors could contribute to the deviation of the predicted values from the reference protein in the validation set. This can be due to variability in the composition among kernels of the same sample, including the spectra, or the analytical error from reference protein content measurements [22,27]. The regression coefficients for the model developed using pre-processing with MSC (Figure 5) show noticeable major peaks at 1143 and 1184 nm. These wavelengths seemed to coincide with protein absorption, similar to those reported in the study of Williams [28]. These could be used for feature selection to develop an instrument with a few wavelengths or for setting a threshold to sort kernels with high or low protein content. The distinct major valleys (negative coefficients) at 1156 and 1499 could be related to water absorption bands as indicated by Peiris et al. [24] and Delwiche, Pitt, and Norris [29], respectively, which may suggest a negative relationship between protein and moisture contents. Yet, this possible negative relationship could not be verified in our study due to the narrow range of moisture content of the samples.

In the prediction of protein content on single kernels in the test set (samples not included in the calibration and validation sets), the results from Table 2 highlight that both SNV and MSC pre-processing could be useful for improving the model performance with an SEP of 0.83 to 0.87% and RPD of 3.24 to 3.40 at 15 factors.

The model with the SG first derivative pre-processing produced the highest SEP at 2.95%. To verify this result, other pre-processing methods involving the SG derivative were explored, including the SG second derivative, SG first derivative + SNV, and SG first derivative + MSC. It was observed that any model with the SG derivative pre-processing would result in a relatively high standard error of prediction, which may suggest that this pre-processing could increase the noise that is inherent to the calibration set or could remove some relevant information during the transformation. This could be related to the wider standard deviation of apparent absorbance at and near 1940 nm (as shown in Figure 3), where SG derivative pre-processing would cause the spectral values to approach zero on the leftmost side of the spectra as a result of smoothing. Pre-processing, such as with the SG derivative, can reveal important peaks relevant to the analyte of interest that will be critical in other studies, but it may not perform well in model development. This finding shows one potential drawback of dependence on pre-processing and on the coefficient of determination as an evaluation metric, as supported in other studies [30]. Future studies may also explore optimizing the SG parameters, including window size and polynomial order, for sorghum applications [31].

**Figure 6:**
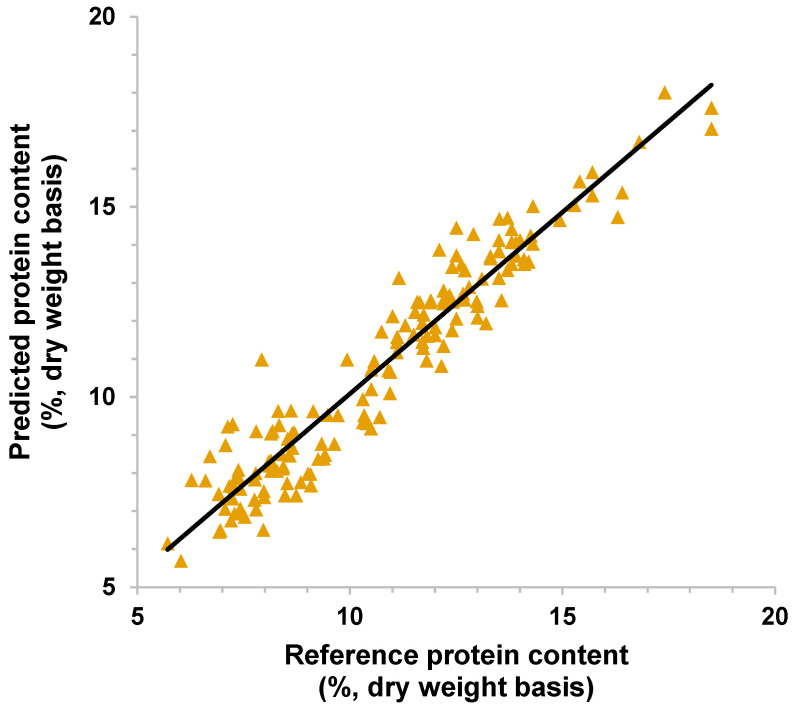
Scatterplot of reference and predicted % protein content in single sorghum kernels on test set samples using MSC as pre-processing.

Using MSC as pre-processing, the scatterplot in Figure 6 shows tight clusters of data points near the line, except for the one kernel predicting a 3% higher protein content than the reference. These metrics may indicate that the developed models have good predictive abilities on single kernels from new or unseen samples. The spread of the data points in the plot demonstrates the wide variability of protein content in kernels from the different samples. The plot also did not reveal signs of any five points glued together, which may pertain to five kernels from the same sample source. In combination with MSC pre-processing, feature extraction and wavelength selection techniques that were found to be effective in other NIR sorghum studies could be potentially explored in improving the model performance and reducing data dimensionality [32].

**Figure 7:**
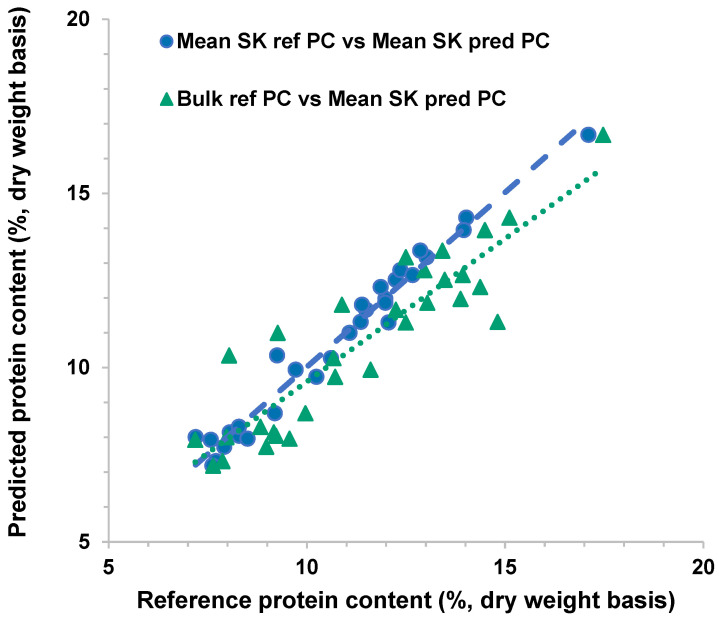
Scatterplot of reference and predicted % protein content (PC) of test set samples using mean single-kernel reference data (round markers) and bulk sample reference data (triangle markers) the lines are the corresponding regression lines.

To compare the single-kernel protein content with the bulk sample protein content (Figure 1) as the reference data for the prediction on test set samples, the mean protein values of the single-kernel data were obtained. In Figure 7, the mean single-kernel reference protein content showed a stronger relationship with the mean single-kernel predicted data than the bulk sample reference protein content. The predicted data seemed to be underestimated or overestimated in some samples when the bulk protein reference data were used. These results support the importance of protein content prediction at the single-kernel level to investigate the composition of individual seeds, which can be useful in sorting and quality control applications.

Overall, the proposed single-kernel NIR approach in our study offers several advantages over other alternative techniques. It requires no sample preparation, such as grinding, thereby saving resources and labor. It is also non-destructive, allowing breeders to save high-protein seeds for future use, which could be useful for early-generation breeding decisions. The NIR spectral range used, as compared to the visible range or short-wave infrared (SWIR) range of other spectrometers, captures the absorption bands related to protein content prediction. Moreover, our study used a different set of kernels from the same sample in calibration for model validation and an independent set of samples for model prediction, minimizing overfitting and testing the model for practical applications. Finally, our method for quality prediction at the single-kernel level covers more sample variability and better kernel resolution than bulk sample analysis. With this approach, breeders and grain processors can evaluate the distribution of protein content within kernels of the same sample.

## 4. Conclusions

In this study, our results showed how single-kernel NIR spectroscopy, in combination with multivariate techniques, could be used for the non-destructive and efficient prediction of protein content in individual sorghum kernels. It was found that either SNV or MSC could be used as a pre-processing method to improve the performance of the protein model developed using partial least squares regression. The calibration model performed comparably well in validation and test sets, signifying its ability to predict kernels with similar characteristics as the calibration set or those from a different set of samples. The identified key wavelengths highlighted the relationship of the spectra to the analytical bands, possibly indicative of protein absorption. These wavelengths could be used for dimensionality reduction, feature selection, thresholding, instrument development, or sorting applications.

To improve the robustness of the model, more sorghum samples with different properties encompassing wider variability (i.e., different cropping years, cultivars, environmental conditions, sizes, colors, or chemical compositions) need to be included in the future. Variable selection using variable importance in projection (VIP) and other methods will also be explored. As the accuracy and precision of the model depend on the reference data, improving the reference method and minimizing the laboratory error will also be vital. To maintain this method, checked samples will be needed for the quality control of the instrument and model.

## Figures and Tables

**Table 1:** PLS regression calibration and validation statistics for different sets of pre-processing.

Pre-Processing	R^2^_C_	SEC	R^2^_V_	SEV	RPD
Raw spectra	0.94	0.72	0.87	1.05	2.69
MSC	0.97	0.51	0.92	0.83	3.40
SG first derivative	0.96	0.59	0.84	1.13	2.50
SNV	0.96	0.53	0.91	0.86	3.28

MSC: multiplicative scatter correction; SNV: standard normal variate; SG: Savitzky–Golay; R^2^_C_: calibration coefficient of determination; R^2^_V_: validation coefficient of determination; SEC: standard error of calibration; SEV: standard error of validation; and RPD: ratio of performance to deviation.

**Table 2:** Prediction performance on the test set samples using different models.

Pre-Processing	R^2^_P_	SEP	RPD
Raw spectra	0.81	1.21	2.33
MSC	0.91	0.83	3.40
SG first derivative	-	2.95	0.96
SNV	0.90	0.87	3.24

MSC: multiplicative scatter correction; SNV: standard normal variate; SG: Savitzky–Golay; R^2^_P_: prediction coefficient of determination; SEP: standard error of prediction; and RPD: relative predictive determinant.

## Data Availability

The original data presented in the study is openly available in Ag Data Commons [https://doi.org/10.15482/USDA.ADC/31316725].

## Abbreviations

The following abbreviations are used in this manuscript:

AACC	American Association of Cereal Chemists
MSC	Multiplicative scatter correction
NIR	Near-infrared spectroscopy
PLSR	Partial least squares regression
RPD	Ratio of performance to deviation
SD	Standard deviation
SEC	Standard error of calibration
SEV	Standard error of validation
SEP	Standard error of prediction
SG	Savitzky-Golay
SKNIR	Single-kernel near-infrared spectroscopy
SNV	Standard normal variate
